# Standardized single-burr-hole aspiration–irrigation with drainage is associated with lower early recurrence in chronic subdural hematoma

**DOI:** 10.3389/fneur.2026.1813950

**Published:** 2026-05-19

**Authors:** Zhenjiang Pan, Xuxu Xu, Shepeng Wei

**Affiliations:** 1 Yangpu District Shidong Hospital of Shanghai, Shanghai, China; 2 Shidong Hospital, University of Shanghai for Science and Technology, Shanghai, China

**Keywords:** aspiration–irrigation, burr-hole craniostomy, chronic subdural hematoma, pneumocephalus, recurrence

## Abstract

**Background:**

Recurrence after burr-hole evacuation for chronic subdural hematoma (cSDH) remains clinically relevant, and modifiable operative details may influence early failure. We evaluated a standardized single-burr-hole aspiration–irrigation with drainage (SBAID) workflow incorporating high-point burr-hole positioning (Wei's point), active aspiration–irrigation, air elimination, and frontal-directed drainage, hypothesizing that improved early cavity–air dynamics would translate into fewer early reoperations.

**Methods:**

In this single-center retrospective cohort study, we included consecutive adults with symptomatic, imaging-confirmed cSDH treated with single-burr-hole evacuation and closed drainage between November 2016 and December 2024. Patients underwent SBAID or conventional single-burr-hole irrigation and drainage (SBID). The primary outcome was recurrence requiring reoperation within 60 days. Secondary outcomes included operative duration, length of stay, postoperative complications, and quantitative imaging markers of early postoperative cavity–air burden (48-h residual subdural space and pneumocephalus volumes, and ~30-day residual cavity volume). Multivariable logistic regression and propensity score–based sensitivity analyses were performed.

**Results:**

Among 186 patients (SBAID, *n* = 151; SBID, *n* = 35), 60-day reoperation-defined recurrence occurred in 2.6% vs. 14.3%, respectively (OR 0.16, 95% CI 0.04–0.64; *P* = 0.013). SBAID was associated with shorter operative time and shorter time to discharge (both *P* < 0.001), smaller 48-h residual subdural space and pneumocephalus volumes, and smaller ~30-day residual cavity volume (all *P* < 0.001). Postoperative seizures were less frequent with SBAID, whereas in-hospital mortality did not differ.

**Conclusions:**

In this single-center retrospective cohort, SBAID was associated with lower early reoperation-defined recurrence and reduced postoperative cavity–air burden vs. conventional SBID.

## 1 Introduction

Chronic subdural hematoma (cSDH) is a common neurosurgical disorder in older adults. Although burr-hole evacuation with closed subdural drainage typically provides rapid decompression, postoperative re-accumulation and recurrence remain clinically important and frequently necessitate rehospitalization and reoperation. Despite the widespread use of standard burr-hole drainage for symptomatic cSDH, the optimal intraoperative workflow remains uncertain, and seemingly minor technical differences can meaningfully influence postoperative cavity dynamics, pneumocephalus, and brain re-expansion—factors that have been consistently linked to recurrence.

Recent randomized evidence has further sharpened attention to operative details. The FINISH trial questioned routine intraoperative irrigation by comparing burr-hole drainage with vs. without irrigation, underscoring the need to identify which specific maneuvers truly improve outcomes (1). In parallel, middle meningeal artery embolization (MMAE) has been explored as an adjunct to reduce treatment failure; however, trial results have been heterogeneous across populations and endpoints, supporting MMAE as an evolving option rather than a replacement for high-quality surgical evacuation in many symptomatic cases (2–4). Collectively, these data reinforce the practical importance of evacuation strategy and air management, as postoperative pneumocephalus and insufficient brain re-expansion are associated with higher recurrence risk (5).

To address these issues, we developed a standardized single–burr-hole aspiration–irrigation with drainage (SBAID) workflow that integrates an anterior high-point burr hole (“Wei's point”), controlled warmed-saline aspiration–irrigation, systematic air elimination, and a predefined frontal-directed drain trajectory. We hypothesized that this air-eliminating workflow would favorably shift early postoperative cavity–air dynamics on quantitative imaging and translate into fewer early reoperation-defined recurrences within 60 days compared with conventional single–burr-hole irrigation and drainage (SBID). Accordingly, we conducted a single-center retrospective cohort study to evaluate this workflow–imaging–outcome linkage.

## 2 Materials and methods

### 2.1 Study design and ethics

We performed a single-center retrospective cohort study of consecutive adults undergoing surgery for symptomatic chronic subdural hematoma (cSDH) at Shidong Hospital from November 2016 to December 2024. The Institutional Review Board approved the study and waived written informed consent because of the retrospective, deidentified design. Reporting followed the STROBE guideline (6).

### 2.2 Participants

Eligible patients were adults (≥18 years) with symptomatic, CT- or MRI-confirmed cSDH evaluated for surgical evacuation. Exclusion criteria were: (1) acute traumatic subdural hematoma requiring craniotomy; (2) subdural empyema; (3) concomitant intracranial lesions requiring additional neurosurgical procedures; (4) missing key perioperative imaging (baseline and/or routine 48-h postoperative CT) or inability to determine 60-day recurrence status; and (5) small, focal collections with minimal mass effect (managed conservatively in our practice). Radiographically septated or multiseptated cSDH was not considered an exclusion criterion, provided that the patient was treated with single-burr-hole evacuation and closed drainage within the study workflows. Consecutive patients treated with single-burr-hole evacuation and closed drainage using either the SBAID or SBID workflow were included. Bilateral cSDH was defined by baseline imaging. In bilateral cases, the same surgical workflow (SBAID or SBID) was applied to both sides during the index procedure. Primary analyses were conducted at the patient level (one index surgery per patient), rather than at the hematoma-side level. Early recurrence was defined as any reoperation within 60 days after the index procedure.

### 2.3 Surgical techniques

Operations were performed by a dedicated neurosurgical team (majority by the senior author). Workflow selection largely reflected protocol adoption over time rather than baseline hematoma features; potential nonrandom allocation was addressed with multivariable adjustment and propensity score–based sensitivity analyses. Because SBAID was increasingly adopted later in the study period, temporal confounding related to evolving perioperative practice and operator experience cannot be fully excluded.

**Figure 1:**
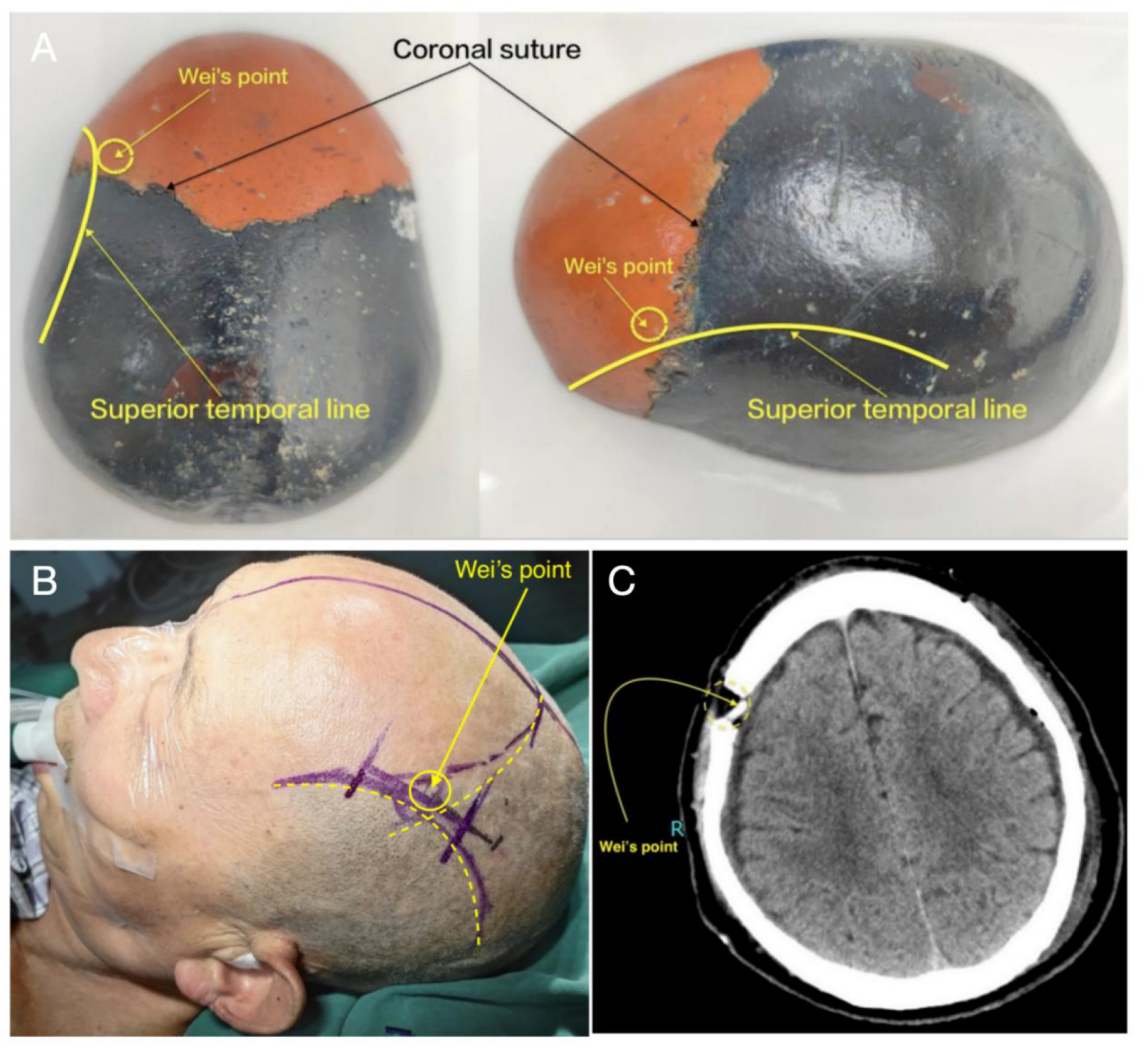
Definition and perioperative identification of Wei's point for burr-hole placement. **(A)** Cadaveric skull specimen demonstrating Wei's point relative to the superior temporal line and coronal suture. **(B)** Preoperative surface marking of Wei's point in a representative patient. **(C)** Postoperative axial CT confirming the burr-hole position at Wei's point. Wei's point is defined as 0.5 cm medial to the superior temporal line and 1 cm anterior to the coronal suture.

**Figure 2:**
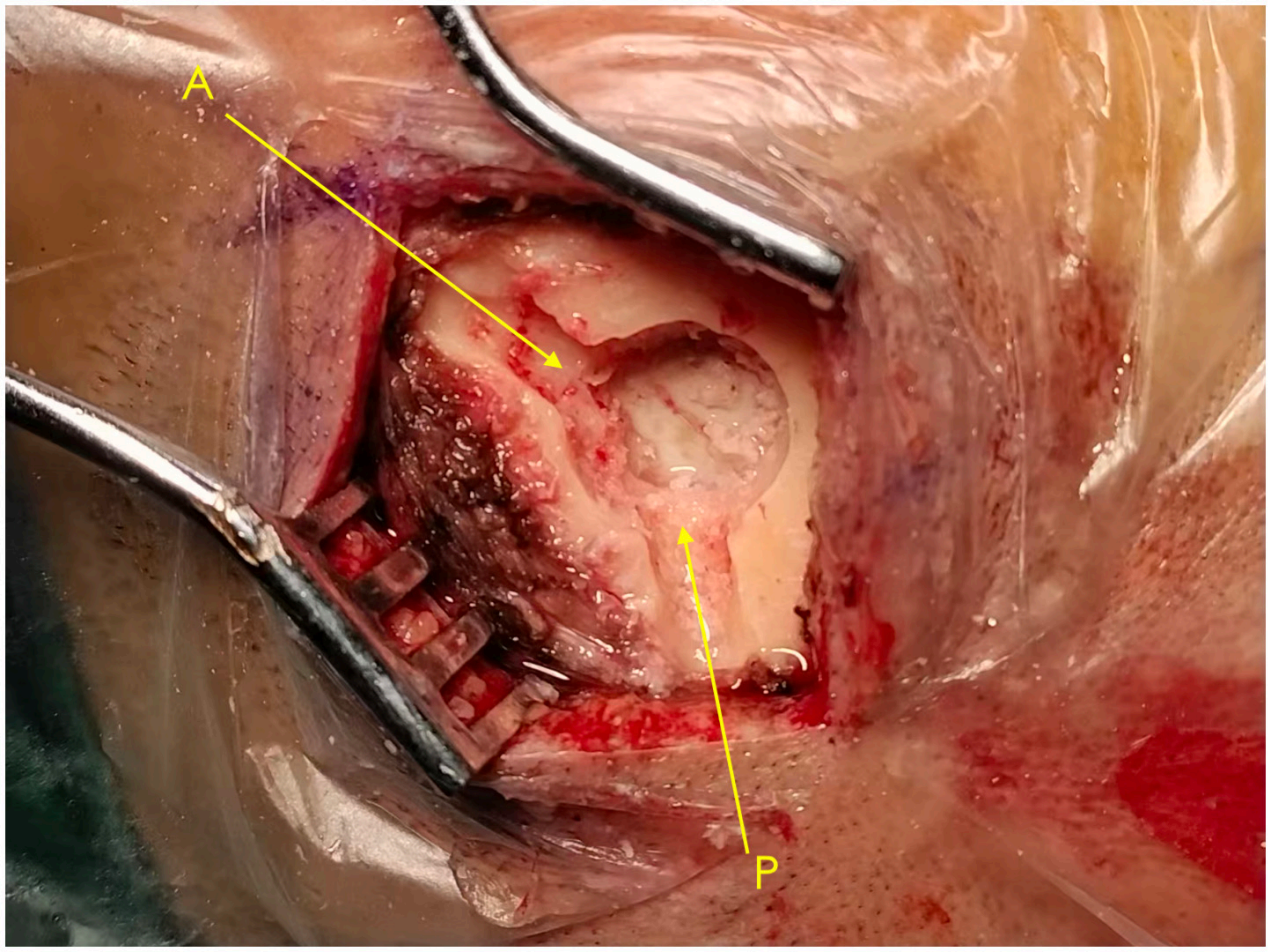
Intraoperative photograph illustrating the anterior **(A)** and posterior **(P)** bony tunnels created from the burr hole during SBAID. The anterior tunnel supports a posteriorly directed trajectory toward the vertex tubercle, whereas the posterior tunnel facilitates an anteriorly directed trajectory toward the frontal pole.

**Figure 3:**
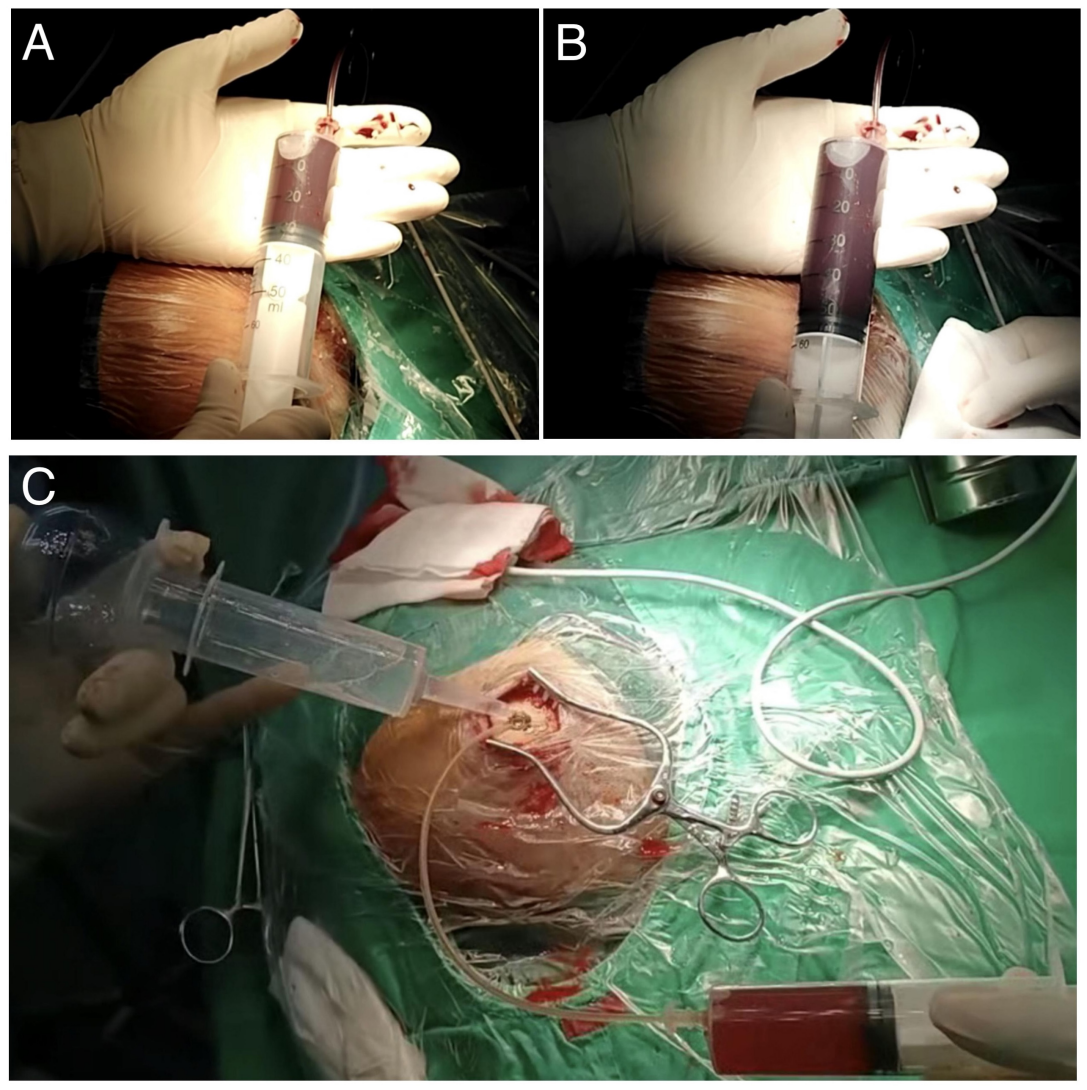
Intraoperative illustration of active aspiration–irrigation in the SBAID workflow. **(A)** Initial aspiration of liquefied hematoma contents through the burr hole using a syringe. **(B)** Continued aspiration with progressive evacuation of dark hematoma fluid, demonstrating ongoing clearance of the subdural cavity. **(C)** Simultaneous irrigation and aspiration: warmed saline is infused through the burr hole while hematoma fluid and residual debris are aspirated through a second syringe, illustrating the active aspiration–irrigation technique used to improve cavity clearance.

SBAID (Single–Burr-Hole Aspiration–Irrigation With Drainage). Standardized steps included: (1) Wei's point burr-hole position (0.5 cm medial to the superior temporal line and 1 cm anterior to the coronal suture) to improve access and facilitate air elimination in the supine position (Figure 1); (2) bone-groove preparation (two shallow anterior/posterior grooves) to create smooth bony tunnels and reduce shear during catheter passage (Figure 2); (3) active aspiration–irrigation using warmed saline (37 °C) with repeated aspiration–irrigation cycles and systematic redirection until effluent cleared (Figures 3A–C); (4) dexamethasone lavage (final 200 mL saline containing dexamethasone sodium phosphate 10 mg); (5) drain placement with the catheter tip directed toward the frontal pole for closed drainage; and (6) systematic air elimination by positioning maneuvers to make the burr hole the highest point and instilling saline to displace residual air before system closure. The active aspiration–irrigation component of the SBAID workflow is illustrated in Figure 3.

SBID (Conventional Single–Burr-Hole Irrigation With Drainage). A burr hole was placed over the region of maximal hematoma thickness (commonly near the parietal eminence). No bone grooves were created. The cavity was irrigated with warmed saline (2,000–3,000 mL) until effluent cleared, without dedicated aspiration. No dexamethasone was added. A subdural catheter was placed for closed drainage.

### 2.4 Postoperative management

Postoperative care was standardized across workflows. Patients were maintained on bed rest with gravity-dependent closed drainage for approximately 48 h without active suction; the drainage bag was kept on the bed to avoid excessive dependent drainage. Supine positioning with a flat head was encouraged when tolerated, and lateral positioning on the affected side was recommended. Routine CT was obtained within 48 h to assess residual cavity volume, pneumocephalus, and brain re-expansion.

Drain output and appearance were monitored. If 24-h output exceeded 100 mL and became clear/transparent, cerebrospinal fluid admixture was suspected and the drain was clamped and/or removed at the treating neurosurgeon's discretion.

Rescue protocol: If re-expansion was inadequate (persistent collection thickness >1.5 cm) with the catheter *in situ*, intralesional urokinase (20,000 IU in 5 mL saline daily) was administered, with 2–4 h of post-instillation clamping, for 1–3 days based on imaging response.

Recurrence management: Repeat evacuation using SBAID was preferred; middle meningeal artery embolization was not routinely performed during the study period.

### 2.5 Data collection and imaging

Clinical, radiographic, and operative variables were abstracted from electronic medical records (demographics, comorbidities, antithrombotic therapy, neurological status, hematoma features, operative details, complications, and outcomes). Because the hematoma cavity was not routinely inspected endoscopically, internal septation was not confirmed intraoperatively. Accordingly, septated or membranous architecture was assessed only on baseline preoperative imaging. No septation-specific fenestration or endoscopic manipulation was performed. CT measurements were performed by an assessor blinded to workflow assignment. Hematoma volume was estimated using the ABC/2 method (7); the 48-h postoperative subdural cavity volume and the ~30-day residual cavity volume were estimated similarly and analyzed separately from intracranial air.

Pneumocephalus volume at 48 h was quantified using semi-automated segmentation in 3D Slicer with thresholding, region-growing where applicable, and manual refinement to exclude extracranial air and artifacts. Reliability was evaluated in a randomly selected subset using repeat segmentation (same and/or second assessor), with agreement assessed by intraclass correlation coefficient and/or absolute volume differences; discrepancies were resolved by consensus.

### 2.6 Outcomes

#### 2.6.1 Primary outcome

Clinically meaningful early recurrence requiring reoperation within 60 days, defined as re-accumulation >10 mm with new or worsening symptoms prompting repeat surgery.

#### 2.6.2 Secondary outcomes

Recurrence over total follow-up; quantitative early postoperative imaging measures (48-h pneumocephalus volume and 48-h residual cavity volume) as candidate surrogates of cavity–air dynamics; ~30-day residual cavity volume; percentage volume reduction; complications (rebleeding, infection, seizures, neurological deterioration); length of stay; mortality; and operative duration.

Follow-up was determined from outpatient visits and repeat CT when available, supplemented by review of institutional readmissions. Total follow-up was the interval from index surgery to last documented clinical contact and/or imaging and is reported as median (IQR). Patients without follow-up beyond discharge were censored at discharge.

### 2.7 Statistical analysis

Continuous variables are reported as mean ± SD or median (IQR), and categorical variables as *n* (%). The patient was the unit of analysis. To avoid within-patient non-independence, bilateral cSDH cases were analyzed at the patient level rather than as separate hematoma sides. Groups were compared using t tests or Mann–Whitney U-tests for continuous variables and χ^2^ or Fisher exact tests for categorical variables, as appropriate. Multivariable logistic regression assessed the association between workflow (SBAID vs. SBID) and early recurrence, adjusting for prespecified covariates (age, sex, antithrombotic therapy, laterality, baseline hematoma volume, density pattern, trauma history, and comorbidities), reported as odds ratios (ORs) with 95% confidence intervals (CIs). Sensitivity analyses included propensity score methods (inverse probability weighting and/or 1:1 matching) (8), restriction to first procedures, and alternative recurrence definitions. Analyses used R (version 4.0.2) and Stata 17; 2-sided *P* < 0.05 was considered statistically significant.

## 3 Results

### 3.1 Cohort and baseline characteristics

A total of 186 consecutive patients with symptomatic chronic subdural hematoma were included (SBAID, *n* = 151; SBID, *n* = 35). Baseline characteristics are shown in Table 1. Patients treated with SBAID were older than those treated with SBID (mean age 72 vs. 65 years, *P* < 0.001). Other baseline demographic and clinical profiles were broadly comparable, including sex distribution, comorbidities, antithrombotic therapy, neurological deficits at presentation, and seizure history. The proportion of bilateral hematomas was similar between groups (17.9% vs. 17.1%). Preoperative CT features—including membranous appearance and density patterns/subtypes—did not differ materially between groups (Table 1).

**Table 1:** Baseline characteristics and preoperative CT features in the SBAID and SBID groups.

Characteristic	SBAID (*n* = 151)	SBID (*n* = 35)	*P-value*	Effect estimate (95% CI)
Age, years	72.0 ± 10.5	65.0 ± 9.5	<0.001	MD 7.00 (3.35–10.65)
Male	91 (60.3%)	23 (65.7%)	0.701	OR 0.79 (0.37–1.71)
History of trauma	119 (78.8%)	22 (62.9%)	0.078	OR 2.20 (1.00–4.84)
History of CVA	61 (40.4%)	11 (31.4%)	0.441	OR 1.48 (0.68–3.24)
Bilateral hematoma	27 (17.9%)	6 (17.1%)	1	OR 1.05 (0.40–2.78)
CT: membranous/septated appearance	40 (26.5%)	8 (22.9%)	0.831	OR 1.22 (0.51–2.90)
Seizure at presentation	14 (9.3%)	2 (5.7%)	0.74	OR 1.69 (0.37–7.78)
Anticoagulant therapy	85 (56.3%)	16 (45.7%)	0.266	OR 1.53 (0.73–3.20)
Neurological deficits	89 (58.9%)	24 (68.6%)	0.34	OR 0.66 (0.30–1.44)

Values are mean ± SD or n (%), unless otherwise indicated. P-values were calculated using Welch's t-test for continuous variables and Fisher's exact test for categorical variables. Effect estimates are presented as mean difference (MD) or odds ratio (OR) with 95% confidence intervals (CI).

CVA, cerebrovascular accident; CT, computed tomography.

### 3.2 Clinical outcomes

#### 3.2.1 Operative metrics

Operative duration was shorter with SBAID than with SBID (52.6± 5.4 vs. 59.5 ± 4.8 min, *P* < 0.001), corresponding to a mean difference of −6.9 min. Time from procedure to discharge was also shorter in the SBAID group (5.9 ± 1.1 vs. 6.8 ± 1.3 days, *P* < 0.001; mean difference −0.9 days) (Table 2).

**Table 2:** Postoperative outcomes and follow-up findings in SBAID and SBID groups.

Outcome	SBAID (*n* = 151)	SBID (*n* = 35)	*P-value*	Effect estimate (95% CI)
Primary outcome				
Recurrence requiring reoperation within 60 days	4 (2.6%)	5 (14.3%)	0.013	OR 0.16 (0.04–0.64)
Clinical outcomes				
Duration of surgery, min	52.6 ± 5.4	59.5 ± 4.8	<0.001	MD −6.90 (−8.75 to −5.05)
Interval from procedure to discharge, days	5.9 ± 1.1	6.8 ± 1.3	<0.001	MD −0.90 (−1.38 to −0.42)
Postoperative seizures	2 (1.3%)	3 (8.6%)	0.047	OR 0.14 (0.02–0.89)
Deaths (in-hospital)	3 (2.0%)	2 (5.7%)	0.237	OR 0.33 (0.05–2.08)
Follow-up				
Follow-up duration, weeks	32.4 ± 7.3	29.4 ± 8.5	0.06	MD 3.00 (−0.13 to 6.13)
Lost to follow-up	18 (11.9%)	5 (14.3%)	0.776	OR 0.81 (0.28–2.36)
Stable cSDH at follow-up^*^	121/133 (91.0%)	27/30 (90.0%)	1	OR 1.12 (0.30–4.25)
Radiographic outcomes				
Residual subdural space volume at 48 h, cm^3^	41.4 ± 7.1	47.4 ± 9.1	<0.001	MD −6.00 (−9.31 to −2.69)
Pneumocephalus volume at 48 h, cm^3^	21.5 ± 5.6	25.6 ± 5.4	<0.001	MD −4.10 (−6.15 to −2.05)
Residual cavity volume at ~30 days, cm^3^	22.4 ± 4.2	27.4 ± 5.7	<0.001	MD −5.00 (−7.06 to −2.94)
Percentage volume reduction at ~30 days, %	73.4 ± 7.2	71.8 ± 8.6	0.313	MD 1.60 (−1.56 to 4.76)
Preoperative hematoma volume, cm^3^	101.2 ± 19.3	101.3 ± 16.3	0.975	MD −0.10 (−6.45 to 6.25)

Values are mean ± SD or n (%), unless otherwise indicated. ^*^Stable cSDH at follow-up was calculated among patients with available follow-up data (SBAID: 121/133; SBID: 27/30). Recurrence refers to reoperation-defined recurrence within 60 days. P-values were calculated using Welch's t-test for continuous variables and Fisher's exact test for categorical variables. Effect estimates are presented as mean difference (MD) or odds ratio (OR) with 95% confidence intervals (CI). For continuous variables, 95% CIs for MD were derived using Welch's t-test (Satterthwaite approximation).

cSDH, chronic subdural hematoma.

#### 3.2.2 Follow-up

Mean follow-up duration was 32.4 ± 7.3 weeks in the SBAID group and 29.4 ± 8.5 weeks in the SBID group (*P* = 0.060). Loss to follow-up occurred in 18 patients (11.9%) and 5 patients (14.3%), respectively.

#### 3.2.3 Primary outcome (early recurrence)

Recurrence requiring reoperation within 60 days occurred in 4 of 151 patients (2.6%) in the SBAID group and 5 of 35 patients (14.3%) in the SBID group (*P* = 0.013), representing an absolute risk reduction of 11.7 percentage points. The odds ratio for early recurrence with SBAID vs. SBID was 0.16 (95% CI 0.04–0.64).

#### 3.2.4 Secondary clinical outcomes

In-hospital mortality occurred in 3 patients (2.0%) in the SBAID group and 2 patients (5.7%) in the SBID group (*P* = 0.237). Detailed case-level information on the five in-hospital deaths is provided in Supplementary Table 1. The causes of death were heterogeneous and included postoperative infection/sepsis, acute postoperative subdural hematoma, and excessive drainage–associated intracranial hypotension. Postoperative seizures were less frequent with SBAID (1.3% vs. 8.6%, *P* = 0.047; OR 0.14, 95% CI 0.02–0.89).

### 3.3 Radiographic outcomes

Baseline preoperative hematoma volume was similar between groups (101.2 ± 19.3 vs. 101.3 ± 16.3 cm^3^, *P* = 0.975). At 48 h, the SBAID group demonstrated a smaller residual subdural space volume (41.4 ± 7.1 vs. 47.4 ± 9.1 cm^3^, *P* < 0.001) and lower pneumocephalus volume (21.5 ± 5.6 vs. 25.6 ± 5.4 cm^3^, *P* < 0.001). At approximately 30 days, residual cavity volume remained smaller with SBAID (22.4 ± 4.2 vs. 27.4 ± 5.7 cm^3^, *P* < 0.001). The percentage reduction in hematoma volume from baseline to ~30 days did not differ between groups (73.4 ± 7.2% vs. 71.8 ± 8.6%, *P* = 0.313). Radiographic and clinical outcomes are summarized in Table 2.

## 4 Discussion

In this single-center retrospective cohort of 186 patients with symptomatic chronic subdural hematoma (cSDH), a standardized single-burr-hole aspiration–irrigation with drainage (SBAID) workflow—incorporating Wei's point positioning, bone-groove preparation, active aspiration–irrigation, dexamethasone lavage, and a frontal-directed drain trajectory—was associated with markedly lower reoperation-defined recurrence within 60 days than conventional single-burr-hole irrigation and drainage (SBID) (2.6% vs. 14.3%; *P* = 0.013; OR 0.16, 95% CI 0.04–0.64). SBAID was also associated with substantially smaller early postoperative pneumocephalus and residual subdural space volumes. Together, these findings reinforce a pragmatic premise that is sometimes obscured in contemporary debates: in cSDH, optimizing and standardizing the primary operation may yield gains comparable in magnitude to those sought through adjunctive strategies.

The recurrence rate observed with SBAID compares favorably with ranges reported after conventional burr-hole evacuation in contemporary syntheses (9, 24). Notably, these outcomes were achieved without routine middle meningeal artery embolization (MMAE). Our data therefore support a stepwise proposition: before escalating to resource-intensive adjuncts, systematic optimization of the core surgical workflow may represent a high-yield, scalable lever for reducing early failure. Nevertheless, given the retrospective design and single-center setting, these associations require cautious interpretation and prospective multicenter validation to establish external reproducibility and to clarify which SBAID elements are essential vs. optional.

### 4.1 Technical innovations and mechanistic rationale

The SBAID workflow was designed to address three modifiable drivers of recurrence: (i) residual hematoma/inflammatory burden, (ii) postoperative pneumocephalus, and (iii) impaired brain re-expansion. Each component targets a specific mechanism.

Burr-hole positioning (Wei's point). Conventional burr holes are often placed at the region of maximal thickness, whereas postoperative air dynamics are governed by geometry and gravity. Wei's point (0.5 cm medial to the superior temporal line and 1 cm anterior to the coronal suture) positions the burr hole at a higher point in the supine patient to facilitate air evacuation, aligning operative geometry with recurrence biology (11). This rationale is consistent with the established association between pneumocephalus and recurrence (12–14).

#### 4.1.1 Bone grooves

Anterior and posterior grooves enable tangential catheter entry, reduce resistance during insertion, and may lessen shear-related cortical or arachnoid irritation—factors that could influence air ingress and early cavity dynamics.

#### 4.1.2 Active aspiration–irrigation

Randomized evidence has sharpened attention to irrigation as a potentially meaningful intraoperative maneuver rather than a purely ritualized step (1). We extend this logic by coupling infusion with controlled aspiration and systematic redirection to improve clearance while aiming to avoid unnecessary disruption of fragile neomembranes.

Frontal-directed drain trajectory. Drain effectiveness must persist during the early re-expansion phase. A frontal-directed trajectory is intended to maintain drainage in regions where re-expansion may lag, thereby reducing dependent residual collections.

Collectively, these steps define SBAID as a mechanism-informed bundle rather than a set of isolated technical tips.

### 4.2 Intraoperative irrigation and local anti-inflammatory lavage

While FINISH provides high-level evidence that irrigation can matter (1), it does not fully resolve how irrigation should be performed. Passive irrigation may inadequately clear dependent debris or inflammatory substrate. Active aspiration–irrigation is intended to enhance exchange efficiency and broaden cavity sampling through repeated cycles.

The addition of dexamethasone to the final lavage is biologically coherent with inflammatory–angiogenic frameworks of cSDH pathophysiology (16). However, systemic steroid trials underscore that biological plausibility does not guarantee net clinical benefit: DEX-CSDH raised concerns regarding adverse events and overall trade-offs (18), and DECSA further highlights the complexity of positioning steroids relative to surgery (17). Local instillation may offer a different therapeutic index than systemic therapy, but its pharmacokinetics and independent clinical effect require prospective confirmation.

### 4.3 Robustness of the association: adjustment and residual confounding

Observational surgical studies warrant methodological restraint. Although we applied multivariable adjustment and propensity score–based sensitivity analyses to mitigate confounding (8), residual confounding cannot be excluded, particularly in era-linked protocol adoption. Unmeasured factors—such as degree of cerebral atrophy, membrane architecture, surgeon learning curve, and nuanced postoperative management—may contribute to the observed associations. Accordingly, our findings should be interpreted as evidence of a strong association consistent with the proposed mechanistic rationale, rather than as proof of causality.

### 4.4 The emerging role of MMAE: evidence, heterogeneity, and appropriate positioning

The rapid growth of MMAE has reshaped the cSDH landscape. Recent randomized trials (EMBOLISE, STEM, and MAGIC-MT) suggest that adjunctive MMAE can reduce treatment-failure endpoints compared with standard management, although effect sizes, endpoint definitions, and statistical certainty vary across studies (2–4). Broader syntheses also support benefit in selected settings (10). These advances are important.

At the same time, several considerations argue for judicious adoption:

① MMAE is an adjunct, not a substitute for optimized evacuation. MMAE targets neomembrane vascular supply but does not provide immediate decompression or remove residual debris. When failure is driven by incomplete clearance, persistent cavity, or pneumocephalus, workflow optimization remains the most direct intervention.

② Endpoints are heterogeneous. Composite “treatment failure” endpoints are not equivalent to reoperation-defined recurrence, limiting direct comparability across trials and complicating translation to everyday surgical decision-making (3, 4).

③ Randomized evidence is not uniformly positive. JAMA 2025 (EMPROTECT) provides an important counterbalance: in postoperative high-risk patients, adjunctive MMAE did not significantly reduce 6-month recurrence compared with control despite a numerically favorable trend (19).

④ Benefit likely interacts with baseline surgical performance. Absolute gains may be larger where baseline recurrence is high, and smaller where standardized techniques already yield low recurrence—implying that MMAE value may depend on the quality of the primary operation.

⑤ Complications, cost, and access matter. Even low-frequency disabling complications carry disproportionate weight in a generally treatable disease, and resource constraints vary widely (2, 3).

Taken together, these considerations support a risk-adapted strategy: prioritize MMAE for selected patients (e.g., recurrence after optimized surgery, high-risk radiological phenotypes, inability to interrupt anticoagulation, or patients unfit for reoperation) rather than routine first-line adjunctive use for all.

### 4.5 Pneumocephalus and the air-elimination paradigm

Postoperative pneumocephalus is among the most consistent modifiable predictors of recurrence (12, 13), with recent data reinforcing its clinical association (5). Mechanistically, trapped air may impede brain re-expansion, preserve the subdural space, and permit continued exudation from inflamed membranes. SBAID treats air elimination as a procedural endpoint rather than a secondary consideration. Because air elimination is low-cost and broadly implementable, it represents a scalable target for quality improvement across diverse practice settings.

### 4.6 Urokinase as a targeted rescue strategy

For patients with inadequate brain re-expansion while the catheter remains *in situ*, intralesional urokinase is used selectively as an imaging-triggered rescue measure to facilitate fibrinolysis and drainage. Retrospective evidence suggests that urokinase instillation may reduce recurrence without major safety signals (15), and mechanistic plausibility is supported by urokinase-mediated fibrin degradation (20). Institutional “exhaustive drainage” strategies incorporating fibrinolytics have also reported favorable outcomes (21). Importantly, urokinase is positioned as targeted rescue rather than universal prophylaxis to balance potential benefit against unnecessary exposure.

### 4.7 Mechanism-guided management of recurrence

When recurrence occurs, we favor repeat evacuation using the SBAID workflow as the initial approach, reserving MMAE for recurrence despite optimized repeat surgery or for clearly high-risk phenotypes where evacuation alone is unlikely to be sufficient. This sequencing is mechanism-oriented: if recurrence reflects persistent cavity, residual debris, pneumocephalus, or suboptimal drain dynamics, repeat optimized surgery directly targets these drivers. MMAE may be most biologically compelling when recurrence reflects ongoing neomembrane exudation despite adequate mechanical evacuation.

### 4.8 Alignment with contemporary evidence

Recent studies and meta-analyses evaluating recurrence predictors and risk scores consistently identify modifiable postoperative factors—particularly pneumocephalus and residual cavity volume—as key determinants of recurrence (22–24). SBAID is explicitly designed to control these variables. Drain duration also influences recurrence. The DRAIN TIME 2 randomized trial reported lower recurrence with longer drainage compared with very short drainage, with limited incremental benefit beyond 24 h (25). Our approximately 48-h strategy is intended to cover the early re-expansion window while maintaining surveillance, recognizing that the optimal duration may be context-dependent. Regarding irrigation temperature, available data suggest no clear recurrence benefit of warmed vs. room-temperature fluid (26), implying that temperature is unlikely to be a dominant driver of recurrence.

### 4.9 Limitations

Several limitations warrant consideration. First, the retrospective, non-randomized design and era-linked protocol adoption raise the possibility of selection bias and temporal confounding (e.g., evolving imaging, perioperative care, and intervention thresholds). In addition, the unequal group sizes and the older age of the SBAID cohort may have introduced further baseline imbalance that could not be fully eliminated despite multivariable adjustment and propensity score–based sensitivity analyses. Moreover, internal septation was assessed radiographically rather than by direct endoscopic inspection, and no septation-specific procedures were performed; therefore, residual confounding related to hematoma architecture cannot be fully excluded. Second, results from a standardized workflow implemented by a dedicated team may not generalize to centers with different case volumes, training, or operative practices. Third, reoperation-defined recurrence—while clinically meaningful—can be influenced by symptom burden, follow-up intensity, and surgeon decision thresholds; thus, detection bias cannot be excluded without blinded adjudication. Fourth, the small number of recurrence events limits precision and may constrain subgroup analyses. Although in-hospital mortality did not differ statistically between groups, the small number of deaths and the heterogeneity of perioperative causes preclude meaningful comparative inference regarding workflow-specific mortality risk. Fifth, SBAID is a bundled intervention, preventing attribution of benefit to individual components. Sixth, longer-term recurrence, functional recovery, and quality-of-life outcomes were not systematically captured. Seventh, we did not evaluate MMAE within this cohort and therefore cannot estimate its incremental benefit or cost-effectiveness beyond optimized surgery. Finally, representative paired pre- and postoperative imaging from the historical SBID cohort could not be consistently retrieved, limiting direct visual case-level comparison between workflows. These limitations support prospective multicenter studies and randomized designs to test reproducibility, component contribution, and interaction with adjunctive therapies.

### 4.10 Clinical implications and future directions

A. Surgical-quality-first framework. Our findings support prioritizing surgical standardization—minimizing residual cavity and pneumocephalus—before focusing on adjunct selection.

B. Stepwise adoption pathway. A pragmatic sequence may include: (1) Wei's point positioning (11); (2) systematic air elimination (12–14); (3) drain trajectory standardization; (4) active aspiration–irrigation informed by randomized evidence (1); (5) bone grooves for reproducible, atraumatic catheter placement; and (6) dexamethasone lavage as hypothesis-generating given systemic steroid trade-offs (16–18).

C. Risk-adapted MMAE strategy. Informed by randomized trials (2–4, 19) and real-world heterogeneity (10), MMAE may be prioritized for recurrence despite optimized surgery, high-risk radiological phenotypes or clinical constraints (e.g., ongoing anticoagulation), patients unfit for or declining reoperation, or centers with persistently high recurrence despite standardization.

D. Conceptual escalation algorithm. Optimized SBAID → imaging-guided rescue (e.g., urokinase) (15, 20, 21) → repeat optimized SBAID for recurrence → MMAE for recurrence despite optimized repeat surgery or for clearly high-risk phenotypes.

E. Research priorities. Key next steps include multicenter reproducibility and cost-effectiveness studies; component or factorial designs to identify essential SBAID elements; risk-stratified randomized trials of optimized SBAID alone vs. SBAID plus MMAE; and longer-term studies incorporating standardized functional and quality-of-life outcomes.

## 5 Conclusions

In this single-center retrospective cohort, a standardized single-burr-hole aspiration–irrigation with drainage (SBAID) workflow—anchored by high-point burr-hole positioning (Wei's point), controlled aspiration–irrigation, systematic air elimination, and frontal-directed drainage—was associated with substantially lower 60-day reoperation-defined recurrence than conventional single-burr-hole irrigation and drainage (SBID) (2.6% vs. 14.3%), alongside more favorable early postoperative cavity–air metrics. These results were achieved without routine middle meningeal artery embolization (MMAE).

Given the nonrandomized design and the potential for selection and temporal confounding, causal inference is not possible. Accordingly, these findings should be interpreted as associative rather than causal and should be validated in prospective multicenter studies before broader adoption of the workflow can be recommended. Nonetheless, the findings support a “surgical-quality-first” framework in cSDH: optimizing and standardizing the primary operation may represent a direct, scalable pathway to reducing early failure.

MMAE may be best positioned as a risk-adapted adjunct—prioritized for selected high-risk patients and for recurrence despite optimized evacuation—rather than as a universal first-line add-on. Intralesional urokinase may serve as a pragmatic, imaging-triggered rescue option when brain re-expansion is inadequate while drainage remains *in situ*.

Future prospective multicenter studies should confirm reproducibility, disentangle the relative contribution of SBAID components, and formally test whether early cavity–air dynamics mediate recurrence, including comparative evaluation of optimized surgery alone vs. optimized surgery plus adjunctive MMAE to inform evidence-based, risk-adapted care pathways for cSDH.

## Data Availability

The raw data supporting the conclusions of this article will be made available by the authors, without undue reservation.
