# Supplementary material for: Standardized single-burr-hole aspiration–irrigation with drainage is associated with lower early recurrence in chronic subdural hematoma
Source: Front Neurol. 2026 May 19;17:1813950. doi: 10.3389/fneur.2026.1813950 (PMC13225953; doi:10.3389/fneur.2026.1813950)
Supplement: Supplementary file 1 [file Table_1.docx]

Supplementary Table 1. Case-level summary of the five in-hospital deaths

| Case | Group | Immediate cause of death | Notes |
| --- | --- | --- | --- |
| 1 | SBAID | Postoperative infection with sepsis | The patient was 85 years old. High fever and coma developed on postoperative day 6. The family declined further treatment, and the patient died on postoperative day 8. |
| 2 | SBAID | Acute postoperative subdural hematoma detected on postoperative day 1 | The patient was 86 years old. The family declined repeat surgery, and the patient subsequently died. |
| 3 | SBAID | Excessive postoperative drainage associated with marked intracranial hypotension and acute subdural hematoma | On postoperative day 2, cerebrospinal fluid drainage exceeded 500 mL. Head CT showed an acute subdural hematoma. The family declined decompressive craniectomy, and the patient subsequently died. |
| 4 | SBID | Acute subdural hematoma detected after drain removal on postoperative day 2 | The patient was 89 years old. A large acute subdural hematoma was detected within 2 hours after drain removal on postoperative day 2. The family declined repeat surgery, and the patient subsequently died. |
| 5 | SBID | Postoperative infection | The patient was 79 years old. High fever, coma, and neck stiffness developed on postoperative day 6. The family declined further treatment, and the patient died on postoperative day 9. |
